# Supplementary material for: Long non-coding RNA GAS5 acts as proliferation “brakes” in CD133+ cells responsible for tumor recurrence
Source: Oncogenesis. 2019 Nov 18;8(12):68. doi: 10.1038/s41389-019-0177-4 (PMC6861230; doi:10.1038/s41389-019-0177-4)
Supplement: Supplementary file 1 — Supplementary Data [file 41389_2019_177_MOESM1_ESM.pdf]

Supplementary methods

1.G6PD Assay

G6PD activity assay was performed on CD133hi and CD133lo cells using the G6PD activity assay kit (Sigma-Aldrich) according to manufacturer's protocol.

2. BrDU incorporation assay

For BrDU incorporation and nucleic acid synthesis in CD133hi and CD133lo cells, cells were treated with 10ug/ml BrDU (BD Biosciences) following Sox2 or GAS5 silencing. Staining for BrDU was done according to manufacturer's instruction.

3. Glucocorticoid Receptor Activity Assay

CD133Hi and controls were seeded in a 24 well plate. Cells were transfected with the Cignal Reporter Plasmids for GRE using a dual luciferase assay system (Qiagen). Appropriate treatment (siGAS5, dexamethasone, etc) was done for 24 hours. The dual luciferase kit (Promega) was used to measure activity using a luminometer. Each sample was treated in duplicate for each plasmid (the negative reporter the GRE reporter).

4. Proliferation Assay

For proliferation, MTT based assay was used. Briefly, cells were plated in 100ul growth medium in a 96-well plate and appropriate treatment was done for 24h,48h or 72h. Following treatment, 10ul CCK-8 reagent was added to each well and incubated at 37^0C for 1h. The plate was read at 450nm in a Molecular Devices spectrophotometer. Data was normalized to time 0 to get proliferation rate.

Supplementary Figure Legends

1. CD133+ cells isolated from KPC tumors proliferate slower than CD133- cells as seen by proliferation assays (A) CD133+ cells (from KPC001) were arrested in G0/G1 phase with a significant increase in the G0 Phase cells, while the CD133- cells progressed to G2/M phase regularly (B) CD133+ cells had an increase in low Pyronin stained population compared to CD133- cells in cells isolated from KPC001 cell lines (C) CD133 cells from KPC001 cell line also had increased expression of CDK4, CDK6 and Cyclin D2.

2. A G6PD activity assay showed that pancreatic cancer cell lines with increased CD133+ population had an increased activity

3. Pancreatic cancer cell line PanC01 and S2VP10 were cultured under hypoxia (A) nutritional stress (B) or chemotherapy (C), GAS5 expression was also increased.

4 Stimulation of GR with dexamethasone in CD133+ cells did not change their GAS5 expression

A.

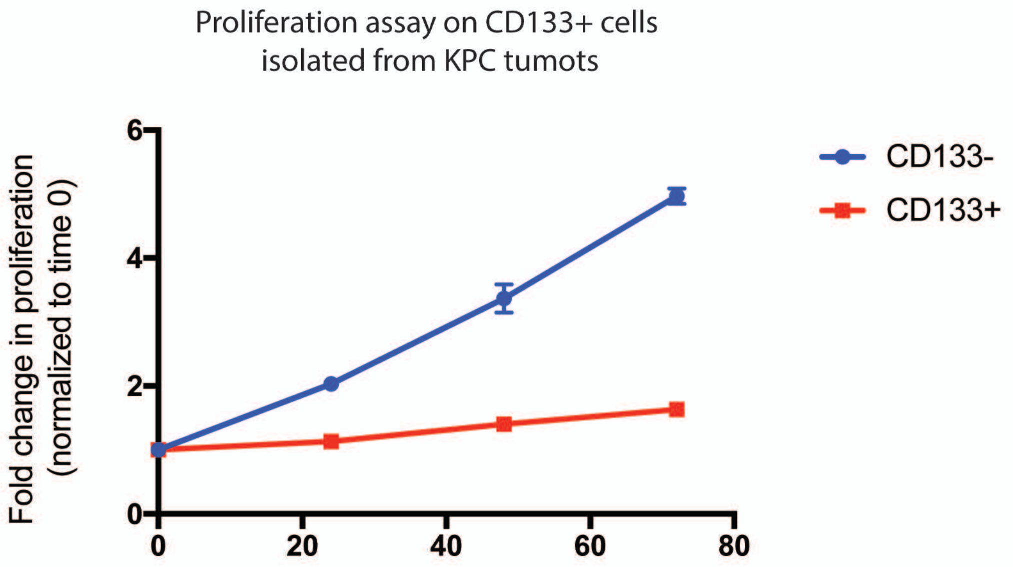

B.

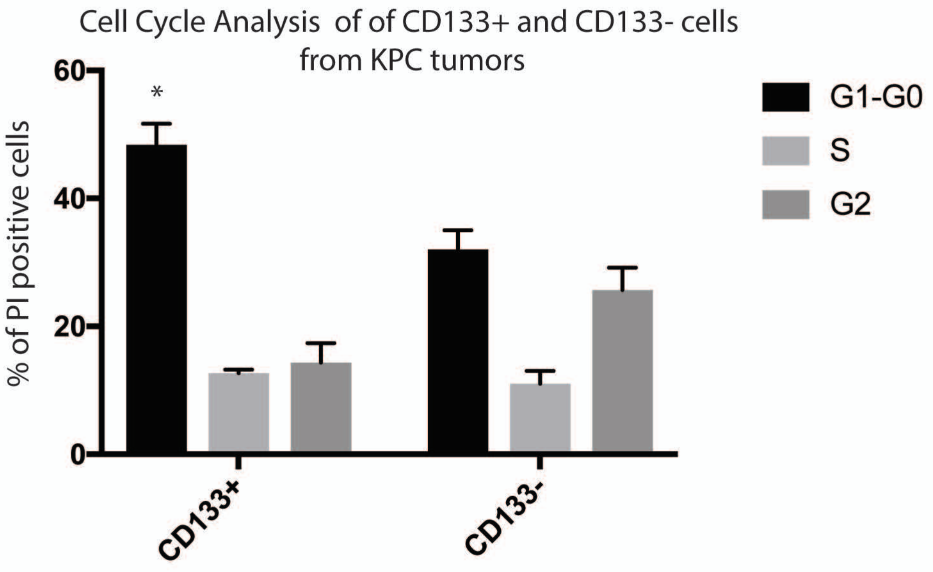

C.

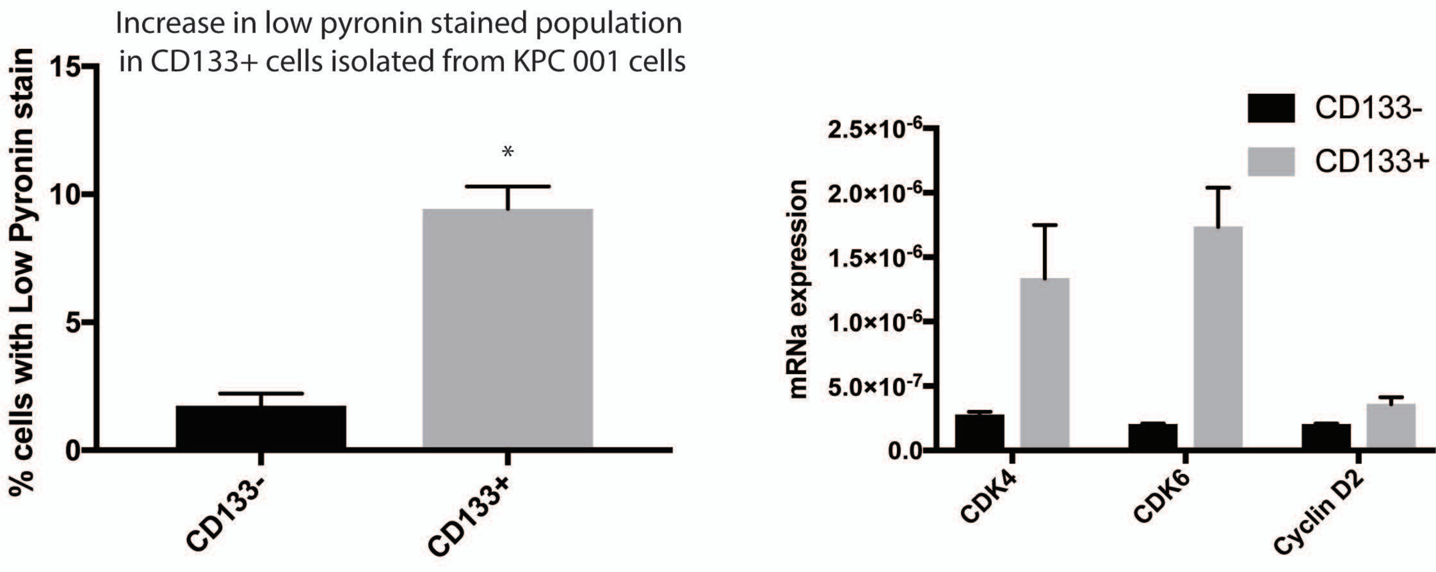

Supplementary Figure 1

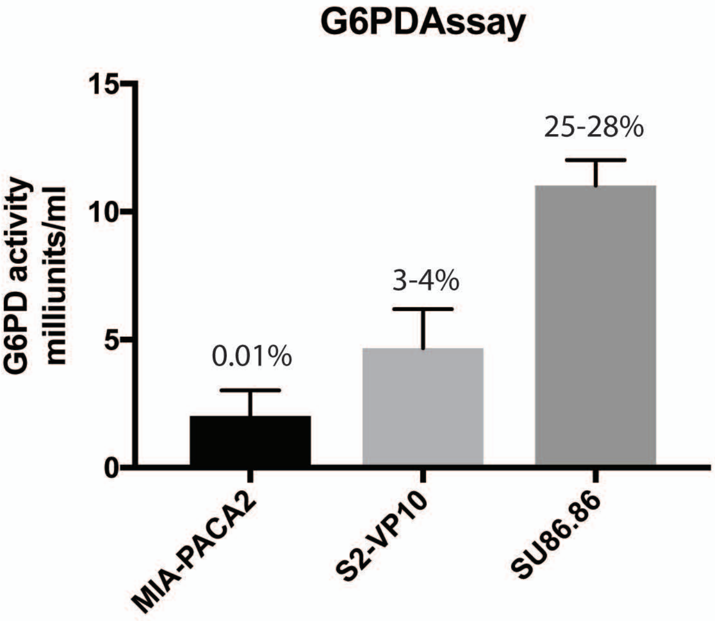

Supplementary Figure 2

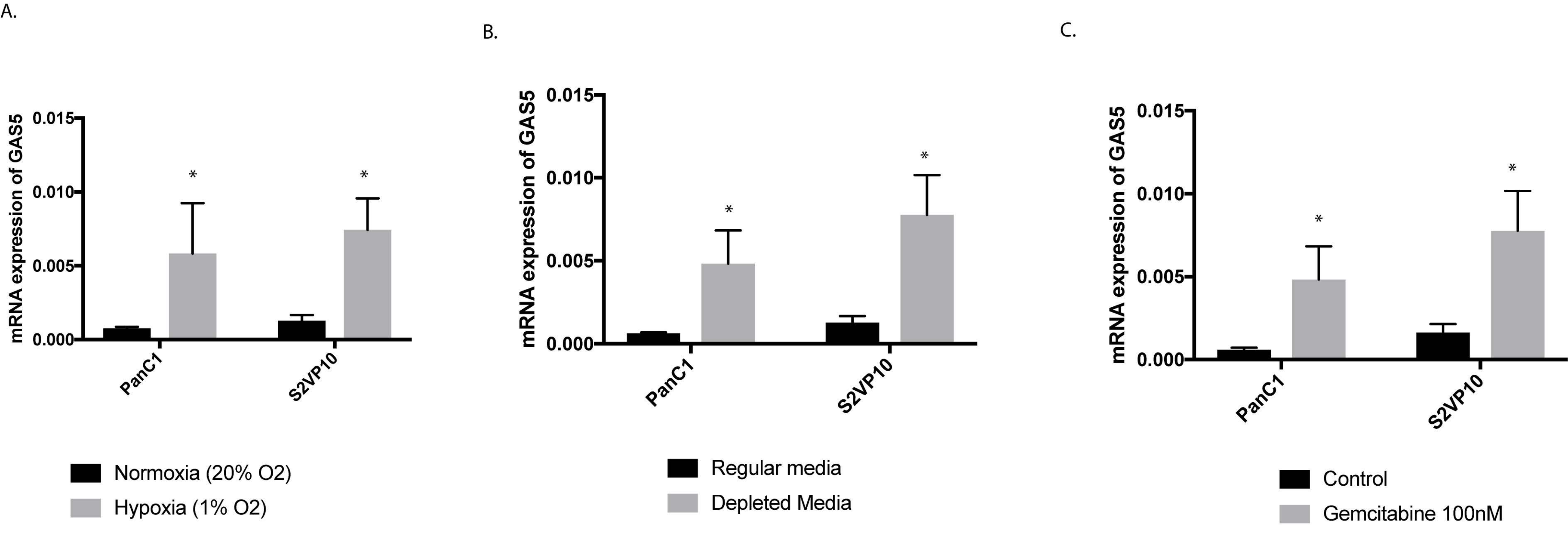

Supplementary Figure 3

Supplementary Figure 3

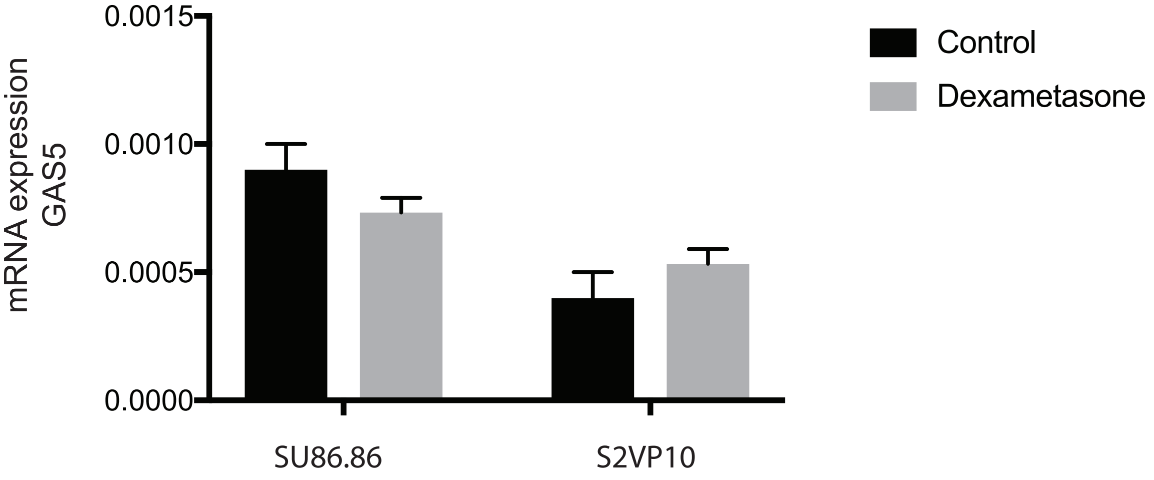

Supplementary Figure 4
